# The ethnic gap in mobility: a comparison of Russian, Somali and Kurdish origin migrants and the general Finnish population

**DOI:** 10.1186/s12889-016-2993-1

**Published:** 2016-04-18

**Authors:** S. Rask, P. Sainio, A. E. Castaneda, T. Härkänen, S. Stenholm, P. Koponen, S. Koskinen

**Affiliations:** National Institute for Health and Welfare, P.O. Box 30, FI-00271 Helsinki, Finland; Deparment of Public Health, University of Turku, Turku, Finland; School of Health Sciences, University of Tampere, Tampere, Finland

**Keywords:** Migrants, Mobility, Ethnic differences, Population-based study

## Abstract

**Background:**

Many ethnic minority populations have poorer health than the general population. However, there is limited knowledge on the possible ethnic gap in physical mobility. We aim to examine the prevalence of mobility limitations in working-age Russian, Somali and Kurdish origin migrants in comparison to the general population in Finland. We also determine whether the association between ethnic group and mobility limitation remains after taking into account socio-economic and health-related factors.

**Methods:**

We used data from the Finnish Migrant Health and Wellbeing Study (Maamu) and the Finnish Health 2011 Survey. The participants comprised 1880 persons aged 29–64 years. The age-adjusted prevalence of difficulties in various mobility tasks was calculated using predictive margins. Logistic regression analysis was used to examine the association between socio-economic, health- and migration-related factors and mobility limitation (self-reported difficulty in walking 500 m or stair climbing). The association between ethnic group and mobility limitation was calculated using logistic regression analysis.

**Results:**

Mobility limitations were much more prevalent among Somali origin women (46 %) and Kurdish origin men (32 %) and women (57 %) compared to men and women in the general Finnish population (5–12 %). In Russian origin men and women, the prevalence of mobility limitation (7–17 %) was similar to the general Finnish population. Socio-economic and health-related factors, but not migration-related factors (time lived in Finland and language proficiency in Finnish or Swedish), were found to be associated with mobility limitation in the studied populations. Somali and Kurdish origin migrants were found to have increased odds for mobility limitation compared to the general Finnish population, even after adjusting for socio-economic and health-related factors (Somalis odds ratio [OR] 3.61; 95 % confidence interval [CI] 2.07–6.29, Kurds OR 7.40; 95 % CI 4.65–11.77).

**Conclusions:**

This study demonstrates a functional disadvantage in Somali and Kurdish origin populations compared to the general Finnish population, even after adjusting for socio-economic and health-related factors. The high prevalence of mobility limitation among Somali origin women and Kurdish origin men and women in Finland demonstrates an acute need to promote the health and functioning of these populations.

## Background

Mobility difficulties are often the first sign of deteriorating functioning and an indicator of pre-clinical stage of disability [1]. Self-reported mobility difficulties also predict mortality [2–4]. Mobility difficulties increase with age, but they are common also among middle-aged adults [5]. Furthermore, a gender gap in mobility has been observed so that women worldwide are more likely than men to report mobility difficulties, with chronic diseases being the main reason for this gap [6].

An ethnic gap is known to exist between ethnic minorities and general populations in many health dimensions. According to international studies, there are differences in self-reported health, morbidity, symptoms and mortality of ethnic minorities and general populations [7–9]. A systematic review on the self-perceived health of migrants in Europe found that most ethnic minority groups are disadvantaged compared to the majority population even after controlling for age, gender, and socio-economic factors [10]. Newcomers are often healthier than their majority population counterparts, but over time this health advantage is reversed, and migrants who have lived in the host country for longer time periods and successive migrant generations have poorer health than majority population peers [7, 11]. The health of migrants in lower socio-economic position is particularly poor [12].

There is a limited amount of research on ethnic differences in physical functioning. Some studies conducted in low and middle income countries have shown that mobility problems appear earlier in life in these populations compared to high income countries [13]. Most studies on this topic have examined African American and Hispanic populations and compared these groups to non-Hispanic Whites in the United States [14–19]. Comparing these results to foreign-born migrants in a European context is in many ways problematic, as ethnic identity and ethnic classification systems are place-, time- and context-specific [20]. In addition, many studies do not provide a clear definition for ethnicity [21].

We are aware of very few studies on the physical functioning of newly migrated populations in Europe. The study of Norrbäck and colleagues [22] demonstrated that mobility disabilities were more common among non-Swedish than Swedish nationals. Lert and colleagues [23] reported a heterogeneous association between ethnicity and functional limitation and reported an increased rate of functional limitations among European-born migrant men and a reduced rate of functional limitations among non-European born migrant men compared to French-born men. According to several studies, factors associated with an increased risk of functional limitations – such as obesity, physical inactivity and poor self-reported health – tend to be more prevalent among migrants than in the general population. For instance, a high prevalence of physical inactivity and obesity has been reported in Iranian and Arabic-speaking migrants in Sweden [24–27] and Somali women in Norway [28]. Similarly, a high prevalence of poor self-reported health was found in Kurdish men and women in Sweden [29].

Given the increase in the proportion and number of migrants and the aging of migrant populations in most affluent countries, reliable information on the functioning of migrants is increasingly important. Population-level information on the prevalence of mobility difficulties is needed for planning health interventions to delay the onset and progression of mobility difficulties. Russian, Somali and Kurdish origin migrants are important migrant groups from a national and an international perspective. Russian-speaking migrants are the largest, Somali origin migrants are the fourth largest, and Kurdish-speaking migrants the sixth largest migrant group in Finland [30]. These three ethnic groups are significant also in other European countries, the United States, Canada, and Australia.

The aim of this study is to: (1) Assess the prevalence of mobility difficulties in Russian, Somali and Kurdish origin migrants in Finland in comparison to the general Finnish population. (2) Determine which socio-economic, health- and migration-related factors are associated with mobility limitation in the studied populations, and ascertain whether the associations differ between the ethnic groups. (3) Determine whether the association between ethnic group and mobility limitation remains after taking into account socio-economic and health-related factors.

## Methods

### Study participants

We use data from the Finnish Migrant Health and Wellbeing Study (Maamu) and the Finnish Health 2011 Survey. In the Maamu Study [31], a sample of 3000 persons of Russian, Somali or Kurdish origin (1000 persons per ethnic group) was randomly selected from the National Population Registry. The sample comprised adults aged 18 to 64 years living in six Finnish cities. The inclusion criteria for Russians were birthplace in the former Soviet Union or Russia and mother tongue Russian or Finnish, for Somalis birthplace in Somalia, and for Kurds birthplace in Iraq or Iran and mother tongue Kurdish. The invitees had resided in Finland for at least one year. In total 70 % of the invited Russian (*n* = 702), 51 % of the Somali (*n* = 512), and 63 % of the Kurdish (*n* = 632) origin persons participated in at least one part of the survey. We use data collected in the health examination, and restrict our sample to persons aged 29–64 years to be able to make comparisons to the general Finnish population. The participation rates in this group were 49 % in Russians (*n* = 362), 39 % in Somalis (*n* = 239), and 58 % in Kurds (*n* = 629).

The Finnish comparison group was selected from the national population-based sample of the Health 2011 Survey [32], including all sampled persons within the same age range and living in the same municipalities as in the Maamu Study (*n* = 2275). Of this sample, 70 % participated in at least one part of the study (*n* = 1582). In this study, we use data collected in the health examination, and exclude participants under the age of 29 because questions on mobility were not asked from the youngest participants in the Health 2011 Survey. The participation rate in this group was 56 % (*n* = 913).

### Data collection

Data in the Maamu Study were collected by trained personnel of Russian, Somali, and Kurdish origin who spoke both the language of the respective target group and Finnish. The study protocol included a face-to-face interview on health and wellbeing and a health examination. A supplementary short interview or questionnaire was collected from those refusing to participate in the long interview. Data from the general Finnish population were collected in the Health 2011 Survey with similar data collection methods and measures. The data used in this study are available from The National Institute for Health and Welfare.

### Ethical approval

The Maamu Study and the Health 2011 Survey were approved by the Coordinating Ethical Committee of the Helsinki and Uusimaa Hospital District, Finland. Written informed consent was obtained from each participant.

### Measures

#### Mobility

As part of the health examination and the short interview/questionnaire, participants were asked questions on mobility. The items included: running a short distance (about 100 m), climbing up several flights of stairs without resting, walking about 500 m, and walking 100 m while carrying a 5 kg bag. The question was formulated as “Can you manage” the respective activity, and the four response categories were: without difficulties, with minor difficulties, with major difficulties, or not at all. Participants reporting any difficulties in a selected item are presented as having difficulties in that activity. Additionally, participants reporting any difficulties in walking 500 m or stair climbing are considered to have mobility limitation, as reported previously by Stenholm and colleagues [33]. The mobility questions are originally based on the recommendation of OECD [34], and they were later modified and complemented for the Health 2000 Survey [35] on the basis of experiences from the Mini-Finland Health Examination Survey [36].

To provide a more comprehensive picture of the physical functioning of the studied populations, we also examined physical performance with the chair stand test [37]. The ability to rise from a chair was tested in the health examination by asking participants to rise from a standard-height chair without using their arms. Those succeeding were asked to rise ten times as fast as possible, measuring the time needed for the performance. Participants who were unable to rise 10 times from the chair or whose test times belonged the lowest tertile (men cut-off > 22.2 s and women > 24.7 s) were defined to have poor performance.

#### Background variables

We included socio-economic, health- and migration-related background variables in our analysis based on previous research demonstrating that age, gender, socio-economic status [38], education, obesity, chronic diseases [39] and time since migration [23] are associated with limitations in mobility and functioning.

The socio-economic variables we used were age, marital status, education, employment status, and economic situation. Age was included as a continuous variable. Marital status was dichotomized into those married or cohabiting and others (including single, separated, divorced, and widowed persons). Education level was dichotomized into high school or higher (or having completed part of high school) and having less than high school education. Employment status was divided into three categories: employed, unemployed, and economically inactive (including e.g., housewives, students, and pensioners). Economic situation was assessed using an item from the WHOQOL-BREF asking if the participant has enough money to meet their needs [40]. The response categories were dichotomized into “not at all or a little” and “moderately, mostly or completely”.

The health-related variables were body mass index (BMI), selected chronic conditions and permanent injury. Based on measured height and weight, we formed a dichotomized variable for BMI (non-obese, <30 kg/m^2^ and obese, ≥30 kg/m^2^). Chronic conditions were self-reports of conditions ever diagnosed by a physician. We formed one dichotomized variable including coronary heart disease, high blood pressure, diabetes, asthma, chronic bronchitis, knee osteoarthritis or hip osteoarthritis, as some of these conditions were too rare to be analyzed separately. Injuries were self-reports of permanent injuries.

The migration-related variables were self-reported time lived in Finland and language proficiency in Finnish or Swedish. Time lived in Finland was divided into three categories: less than 6 years, 6 to 14 years and more than 14 years. Proficiency in the Finnish or Swedish language (the two official languages of Finland) was used as a proxy for integration to the Finnish society. Language proficiency was assessed by asking how well the participant understands Finnish/Swedish (dichotomized into not at all or poorly and moderately or well).

### Statistical analysis

To calculate age-adjusted prevalence rates for each ethnic group, predictive margins were used [41]. The descriptive statistics were calculated using linear or logistic regression analysis, including each determinant separately as the dependent variable and age and ethnic group*gender as the independent variables. The prevalence rates of mobility difficulties were calculated using logistic regression analysis, including age, gender and ethnic group*gender as the independent variables.

The associations between selected socio-economic, health- and migration-related determinants and mobility limitation were calculated using logistic regression analysis, including age, gender and each background variable separately in the model. We examined the second degree interactions (determinant*migrant group*gender) and found these to be statistically non-significant (a *p*-value of < 0.05 was considered statistically significant). The sample size was, however, inadequate to test the second degree interactions for employment status and time lived in Finland. The first degree interactions (determinant*gender) were all statistically non-significant, except for marital status (*p*-value 0.029) and body mass index (*p*-value 0.033). Further investigations on the magnitude of these associations suggested that stratification by gender was not necessary, and thus the main results are presented for men and women together. Descriptive statistics and the prevalence rates of mobility limitation are, however, presented by gender to provide comprehensive information on the level of mobility limitation in the studied populations. The first degree interactions (determinant*migrant group) were all statistically non-significant, except for the variables education (*p*-value 0.049) and economic situation (*p*-value 0.006). The associations between these two variables and mobility limitation are presented by ethnic group.

The association between ethnic group and mobility limitation and the role of socio-economic and health-related factors in that association was examined using logistic regression analysis. In Model 1, age and gender were adjusted for. In Model 2, marital status, education, employment status, and economic situation were added to Model 1. Model 3 included the same variables as Model 2, with the addition of body mass index, selected chronic conditions, and permanent injuries. All results are presented as odds ratios with 95 % confidence intervals.

All analyses were conducted using SAS 9.3/SUDAAN 11.0.0 software, which takes into account the sampling design. Inverse probability weights (IPW) [42] calculated with age group, gender, ethnic group, municipality and marital status were used to reduce bias due to non-response and produce estimates for means and percentages that are representative of Russian, Somali, and Kurdish migrants in Finland. The population sizes were relatively small, and a significant proportion of the total population was included in the sample, and thus the finite population correction [43] was applied in all analyses.

## Results

The main characteristics of the study population are presented in Table 1.

**Table 1:** Descriptive statistics of the study population (29–64 years) by gender

	MEN (*n* = 810)								WOMEN (*n* = 1070)							
Characteristics	Russian (*n* = 124)		Somali (*n* = 88)		Kurdish (*n* = 192)		Finnish (*n* = 406)		Russian (*n* = 238)		Somali (*n* = 151)		Kurdish (*n* = 174)		Finnish (*n* = 507)	
	%^a^	(n)^b^	%^a^	(n)^b^	%^a^	(n)^b^	%^a^	(n)^b^	%^a^	(n)^b^	%^a^	(n)^b^	%^a^	(n)^b^	%^a^	(n)^b^
Age, years (mean)	43.7		40.4		40.6		45.9		45.8		41.4		40.3		45.7	
Married or cohabiting	77.7	(96)	81.8	(68)	83.6	(154)	69.4	(296)	60.0	(149)	70.6	(95)	75.8	(136)	69.3	(346)
High school graduate^c^	79.9	(92)	54.0	(38)	44.9	(82)	55.0	(228)	85.0	(197)	12.8	(18)	38.8	(68)	68.4	(342)
Employment status																
Employed	65.0	(75)	37.3	(31)	42.6	(80)	77.0	(308)	51.9	(122)	15.2	(21)	29.9	(56)	77.6	(391)
Unemployed	30.0	(37)	38.8	(30)	37.5	(64)	5.6	(25)	29.0	(66)	31.9	(38)	31.7	(50)	3.0	(18)
Economically inactive^d^	5.0	(9)	23.8	(21)	19.9	(38)	17.4	(64)	19.1	(48)	52.9	(74)	38.4	(67)	19.3	(94)
Difficult economic situation^e^	22.9	(24)	59.6	(34)	47.4	(81)	8.8	(33)	31.1	(63)	42.3	(37)	41.9	(65)	12.0	(58)
Obese (BMI ≥ 30)^f^	16.4	(19)	5.2	(8)	20.8	(36)	20.4	(70)	20.9	(51)	49.0	(69)	31.0	(48)	20.9	(113)
At least one of the selected chronic conditions^g^	24.8	(37)	13.1	(10)	40.3	(66)	25.4	(129)	28.7	(74)	43.3	(46)	49.5	(77)	30.4	(175)
Permanent injuries	16.6	(20)	7.8	(29)	27.6	(5)	9.3	(7)	12.9	(46)	6.0	(22)	14.5	(35)	4.5	(25)
Time lived in Finland, years																
< 6	26.9	(31)	12.6	(16)	12.1	(24)	NA		19.4	(39)	13.3	(27)	12.7	(24)	NA	
6–14	45.4	(53)	31.9	(27)	62.2	(112)			42.0	(103)	36.2	(49)	57.7	(99)		
> 14	27.7	(37)	55.5	(39)	25.7	(46)			38.6	(94)	50.5	(62)	29.6	(51)		
Poor language proficiency^h^	19.7	(27)	8.5	(13)	12.4	(25)	NA		7.8	(25)	38.3	(55)	20.9	(34)	NA	

NA = Data not available

^a^Age-adjusted and weighted prevalence for all other variables except mean for age

^b^Crude n

^c^Has completed high school or part of high school in any country

^d^Includes e.g., students, housewives and pensioners

^e^Those having not at all or a little money to meet their needs

^f^BMI = Body mass index (kg/m^2^)

^g^Those reporting at least one of the following diseases ever diagnosed by a physician: coronary heart disease, high blood pressure, diabetes, asthma, chronic bronchitis, knee osteoarthritis or hip osteoarthritis

^h^Language proficiency in Finnish or Swedish

### The prevalence of mobility difficulties

In Kurdish origin men and women, the prevalence of all mobility difficulties was significantly higher compared to the general Finnish population (Table 2). Similarly in Somali origin women, all mobility difficulties were more prevalent compared to women in the general Finnish population. In Somali origin men, difficulties in climbing several flights of stairs and mobility limitation, defined as difficulties in walking 500 m or stair climbing, were more common than in men in the general Finnish population. In Russian origin men and women, the prevalence of all mobility difficulties was similar to the general Finnish population. In all the studied migrant groups, poor performance in the timed chair stand test was more common than in the general Finnish population.

**Table 2:** Indicators of mobility in the migrant groups and the general Finnish population^c^ (%)

	Men								Women							
	Russian		Somali		Kurdish		Finnish		Russian		Somali		Kurdish		Finnish	
	%^c^	(n)^d^	%^c^	(n)^d^	%^c^	(n)^d^	%^c^	(n)^d^	%^c^	(n)^d^	%^c^	(n)^d^	%^c^	(n)^d^	%^c^	(n)^d^
Prevalence of difficulties																
walking 500 m	4.1	(4)	3.7	(4)	21.3^***^	(35)	1.7	(6)	4.4	(10)	14.4^***^	(18)	35.4^***^	(53)	3.4	(18)
carrying 5 kg for 100 m	0.0	(0)	7.0	(7)	19.9^***^	(32)	2.8	(13)	8.4	(24)	20.7^**^	(31)	48.1^***^	(73)	9.2	(48)
climbing several flights of stairs	5.3	(8)	12.7^**^	(13)	29.1^***^	(46)	4.7	(27)	15.5	(41)	45.8^***^	(64)	55.4^***^	(87)	11.7	(68)
running 100 m	12.5	(16)	14.8	(16)	33.0^***^	(51)	8.7	(37)	19.8	(51)	46.1^***^	(69)	54.2^***^	(83)	15.7	(89)
Mobility limitation^a^	6.5	(9)	14.8^**^	(14)	31.7^***^	(51)	5.4	(30)	16.7	(44)	46.1^***^	(65)	57.0^***^	(90)	12.0	(69)
Poor performance in chair stand test^b^	32.2^***^	(39)	66.5^***^	(53)	60.0^***^	(100)	16.7	(89)	29.6^***^	(71)	72.5^***^	(105)	54.9^***^	(80)	14.5	(81)

*p-value < 0.05; **p-value < 0.01; ***p-value < 0.001 (difference compared to the Finnish reference group, Satterthwaite adjusted F-statistic)

^a^Self-reported difficulties in walking 500 m or stair climbing

^b^Including chair stand test times belonging to the lowest tertile (men time > 22.2 s and women > 24.7 s) and those unable to rise 10 times from the chair

^c^Age-adjusted and weighted prevalence

^d^Crude n

### Factors associated with mobility limitation

Factors which increased the odds for mobility limitation in all the studied populations were age, being unemployed or economically inactive, obesity, chronic conditions, and injuries (Table 3). The association between education, economic situation and mobility limitation was different between the ethnic groups. A low level of education increased the odds for mobility limitation in all other groups except for Russian origin migrants, while a difficult economic situation increased the odds for mobility limitation only in the general Finnish population. Migration-related factors (time lived in Finland and language proficiency in Finnish or Swedish) did not show an association with mobility limitation.

**Table 3:** Factors associated with mobility limitation^a,b^

	OR (95 % CI)
Age, years	**1.09 (1.07–1.10)**
Marital status	
Married or cohabiting	1.00
Other^c^	1.38 (0.99–1.91)
Education^*^	
Secondary school or less	Russian: 1.24 (0.55–2.78)
	Somali: **6.56 (2.34–18.36)**
	Kurdish: **1.58 (1.01–2.47)**
	Finnish: **2.55 (1.55–4.20)**
High school	1.00
Employment status	
Working	1.00
Unemployed	**1.83 (1.26–2.66)**
Economically inactive^d^	**2.14 (1.53–2.98)**
Economic situation^*^	
At least moderate	1.00
At least quite difficult	Russian: 1.49 (0.73–3.04)
	Somali: 0.87 (0.52–1.80)
	Kurdish: 1.41 (0.89–2.23)
	Finnish: **4.66 (2.58–8.42)**
BMI^e^	
< 30 kg/m^2^	1.00
≥ 30 kg/m^2^	**2.74 (2.05–3.67)**
Selected chronic conditions^f^	
No chronic conditions	1.00
At least one condition	**2.37 (1.79–3.15)**
Permanent injury	
No injuries	1.00
At least one injury	**2.67 (1.78–4.00)**
Time lived in Finland^g^, years	
< 6	1.24 (0.75–2.06)
6–14	0.90 (0.62–1.30)
> 14	1.00
Language proficiency^g^	
Good or fair	1.00
Poor or not at all	1.30 (0.84–2.02)

OR = odds ratio

Bolded ORs represent statistically significant associations

95 % CI = 95 % confidence interval

^*^The first degree interactions (determinant*migrant group) were statistically significant for the variables education (*p*-value 0.049) and economic situation (*p*-value 0.006)

^a^All models are adjusted for age and gender, odds ratio derived from logistic regression models including each background variable separately

^b^Mobility limitation defined as self-reported difficulties in walking 500 m or stair climbing

^c^Includes single, divorced or separated, and widowed persons

^d^Includes e.g., students, housewives and pensioners

^e^Body mass index (kg/m^2^)

^f^Selected chronic conditions include the following self-reported diseases ever diagnosed by a physician: coronary heart disease, high blood pressure, diabetes, asthma, chronic bronchitis, knee osteoarthritis, hip osteoarthritis

^g^Finnish reference group not included in the analysis

### The ethnic gap in mobility

Somali and Kurdish origin migrants were found to have increased odds for mobility limitation compared to the general Finnish population (Table 4). These associations remained statistically significant when adjusting for age, gender, marital status, education, employment status, and economic situation (Model 2). Adjusting additionally for health-related variables (Model 3) made little difference to the associations.

**Table 4:** Association between ethnic group and mobility limitation^a^

	Model 1	Model 2	Model 3
	OR (95 % CI)	OR (95 % CI)	OR (95 % CI)
Finnish	1.00	1.00	1.00
Russian	1.40 (0.92–2.13)	1.47 (0.93–2.32)	1.51 (0.93–2.43)
Somali	**6.31 (4.10–9.71)**	**3.29 (1.96–5.51)**	**3.61 (2.07–6.29)**
Kurdish	**12.25 (8.25–18.19)**	**8.31 (5.31–13.01)**	**7.40 (4.65–11.77)**

OR = odds ratio (derived from logistic regression models)

Bolded ORs represent statistically significant associations

95 % CI = 95 % confidence interval

Model 1: adjusted for age and gender

Model 2: adjusted for age, gender, marital status, education, employment status, and difficult economic situation

Model 3: adjusted for age, gender, marital status, education, employment status, difficult economic situation, body mass index, selected chronic conditions, and permanent injuries

^a^Mobility limitation defined as self-reported difficulty in walking 500 m or stair climbing

## Discussion

This study demonstrates that mobility difficulties are highly prevalent and much more common in Somali origin women and Kurdish origin men and women compared to the general Finnish population. Socio-economic and health-related factors, but not time lived in Finland or language proficiency in Finnish or Swedish, were found to be associated with mobility limitation in the studied populations. Somali and Kurdish origin migrants were found to have increased odds for mobility limitation compared to the general Finnish population, even after adjusting for socio-economic and health-related factors.

Our study adds to literature demonstrating a functional disadvantage in migrant populations. Previous studies have also demonstrated inequalities in mobility between educational and socio-economic groups [38, 39]. In line with these findings, we found that socio-economic factors, including education, employment status and economic situation, are associated with mobility limitation. There were, however, differences in the association of education, economic situation and mobility limitation between the ethnic groups. An important finding of our study was that adjusting for socio-economic factors could not explain the ethnic differences in mobility. This finding is supported by some studies [44], while others have demonstrated that health disparities between ethnic minorities and general populations disappear after controlling for socio-economic status [45].

Our findings on the high prevalence of mobility limitation in Kurdish migrants and Somali origin women are supported by previous studies on the health of these populations. A high prevalence of poor self-reported health was found in Kurdish men and women in Sweden [29]. Similarly, a high prevalence of physical inactivity and obesity has been reported in Iranian and Arabic-speaking migrants in Sweden [24–27] and Somali women in Norway [28]. In the US, foreign-born Arabs, including persons of Iraqi and Somalian origin, were found to be more likely to report functional limitation – meaning a condition that substantially limits one or more basic activities such as walking, climbing stairs, or carrying – compared to White Americans [46]. The reported prevalence rate of functional limitation among persons born in Iraq was 44 % [46], which is comparable with the prevalence rates of mobility limitation in Kurdish origin persons in this study.

**Fig. 1:**
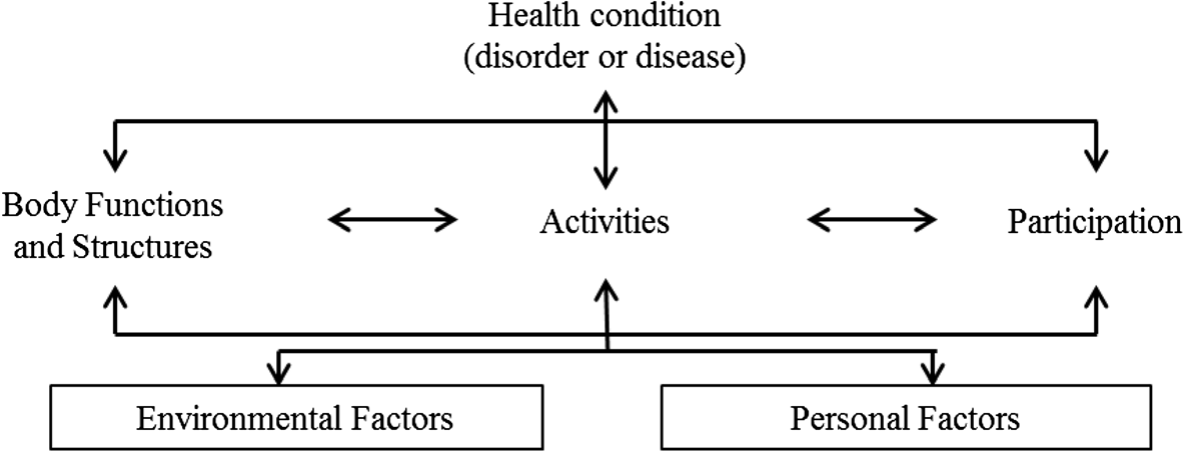
Interactions between the components of ICF [47]

To understand the public health implications of our results, it is necessary to discuss what mobility limitation in working-age migrants may indicate. The International Classification of Functioning, Disability & Health (ICF) conceptualizes a person’s level of functioning as a dynamic interaction between an individual’s health conditions, environmental factors, and personal factors (Fig. 1) [47]. Mobility is classified under ICF’s component activities and participation [48].

As conceptualized in the ICF, there is a relationship between health conditions, body functions (including mental functions) and activities and participation [47]. In line with this theory as well as previously reported findings on the association between chronic conditions, obesity and mobility limitation [39, 49], we found that obesity and chronic conditions were strongly associated with mobility limitation. Health behavior is classified under ICF’s component personal factors. An interactive relationship exists between personal factors, such as physical inactivity, and activities and participation, including mobility [47]. Previous studies confirm that unhealthy life style factors are significant risk factors for incident mobility limitation [50–52]. The combination of mobility disability and obesity has been found to be particularly harmful and have a negative effect also on social capital [22]. In our previous work, we have demonstrated an association between mental health symptoms and mobility limitation in the studied migrant groups [53]. These findings together with the conceptual theory from the ICF demonstrate the complexity of functioning, and suggest that interventions to delay the onset and progression of mobility difficulties in the studied populations should be multi-dimensional.

### Strengths and limitations

Important strengths of our study are the population-based study design and the relatively high participation rate compared to other migrant health studies. Other merits are that we have analyzed three migrant groups separately and compared the migrant groups to the general population. There are also some limitations. The sample size of the study is relatively small. Although the participation rate was satisfactory, it is generally known that the effects of non-response cannot be completely corrected for, particularly for Somalis among whom the participation rate was lowest. Moreover, the findings of this study may not be generalizable outside Russian, Somali and Kurdish populations in Finland. In men, reporting mobility difficulties may be considered socially undesirable, which may be a source of reporting bias. It is also known that self-reported activity limitations may be inaccurate if the respondent does not routinely undertake the requested activities [17]. Although the included items on mobility can be considered very basic activities, not routinely undertaking these activities may apply to Somali and Kurdish origin women resulting in overestimated difficulties. However, poor performance in the chair stand test was also very common in these groups, supporting the self-reported results. Instead, in Russian origin migrants, the prevalence of self-reported mobility difficulties did not differ from the general Finnish population, but poor performance in the chair stand test was more common than in the general Finnish population. This may be a demonstration of both cultural differences and differences in the assessment situation leading to the participant being more or less eager to demonstrate their capacity to rise from the chair. It is also important to note that the included background variables are partial, and other factors, such as type of migration, are also known to influence the health of migrants [54]. Lastly, no causal relations can be shown due to the cross-sectional study design.

## Conclusions

This study demonstrates a functional disadvantage in Somali and Kurdish origin populations compared to the general Finnish population, even after adjusting for socio-economic and health-related factors. The high prevalence of mobility limitation particularly among Somali origin women and Kurdish origin men and women demonstrates an acute need to promote the health and functioning of these populations in Finland. Due to the cross-sectional study design, the causes of mobility limitation in the studied migrant groups cannot be addressed in this study. Future research and longitudinal studies are needed to clarify the pathways through which ethnic minority status is associated with mobility limitation and to confirm these results in other migrant populations.

### Ethics and consent to participate

The Maamu Study and the Health 2011 Survey were approved by the Coordinating Ethical Committee of the Helsinki and Uusimaa Hospital District, Finland. Written informed consent was obtained from each participant.

### Consent to publish

Not applicable.

### Availability of data and materials

The dataset supporting the conclusions of this article is available upon request from The National Institute for Health and Welfare (THL). Guidelines are available at https://www.thl.fi/en/web/thlfi-en/research-and-expertwork/population-studies/migrant-health-and-wellbeing-study-maamu-/information-for-researchers.
